# Supplementary material for: An unconventional proanthocyanidin pathway in maize
Source: Nat Commun. 2023 Jul 19;14:4349. doi: 10.1038/s41467-023-40014-5 (PMC10356931; doi:10.1038/s41467-023-40014-5)
Supplement: Supplementary file 1 — Supplementary Inforamtion [file 41467_2023_40014_MOESM1_ESM.pdf]

Fig. S1

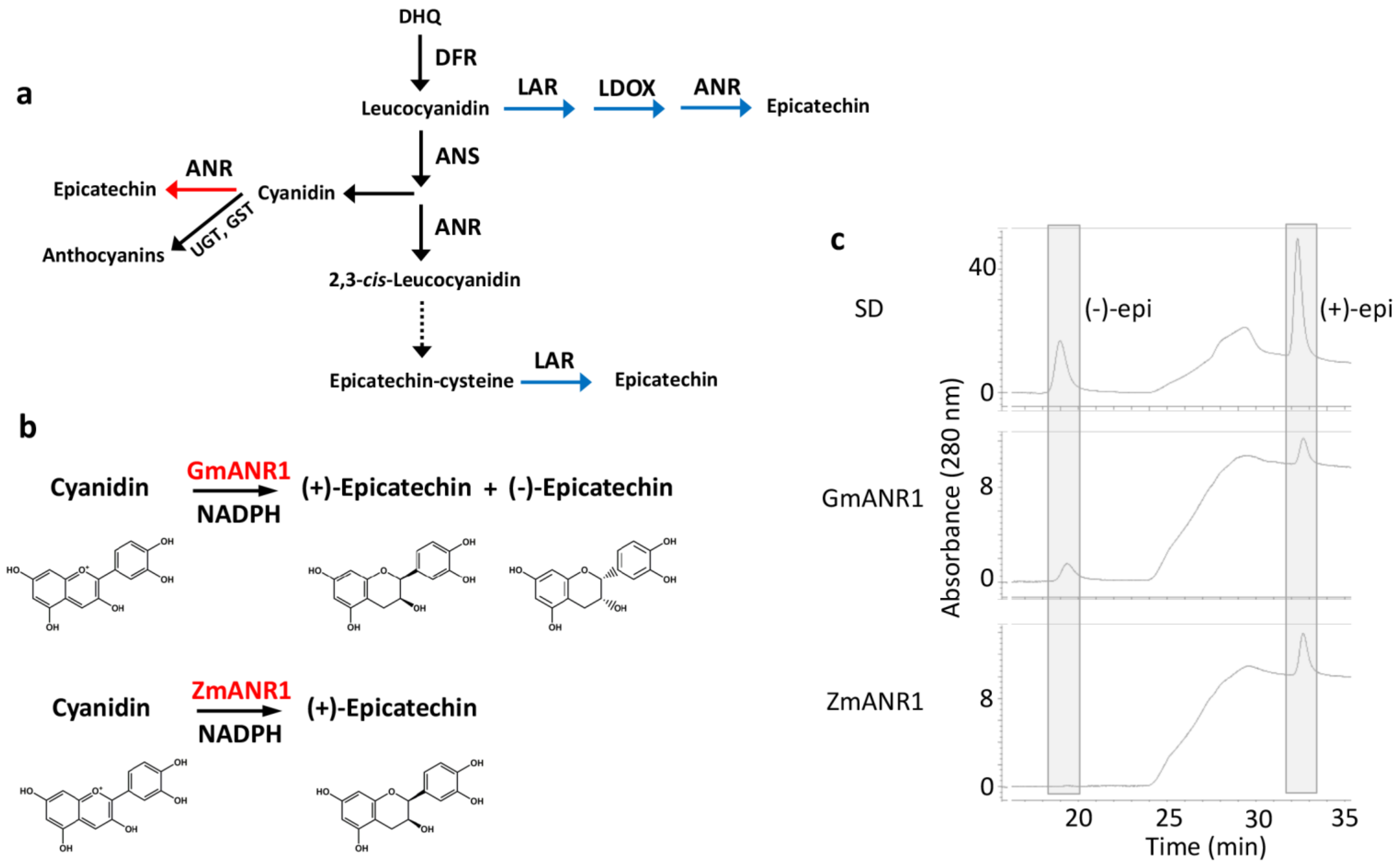

Fig. S1 ANR enzymes from maize and soybean produce different epicatechin stereoisomers *in vitro*. (a) Simplified PA and anthocyanin biosynthesis pathways in plants. Red arrow indicates the reaction catalyzed by ANR existing in both maize and soybean, and blue arrows indicate reactions found in soybean but not in maize. DHQ, dihydroquercetin; DFR, dihydroflavanol 4-reductase; ANS, anthocyanidin synthase; ANR, anthocyanidin reductase; UGT, UDP-glucose: flavonoid glucosyltransferase; GST, glutathione S-transferase; LAR, leucoanthocyanidin reductase; LDOX, leucoanthocyanidin dioxygenase. Some species have a pathway from leucocyanidin to epicatechin starter units that features a LDOX (related to ANS) (23). This pathway is absent in Arabidopsis (b) GmANR1 and ZmANR1 produced different epicatechin stereoisomers *in vitro*. *GmANR1* and *ZmANR1* were cloned from *Glycine max* (cv *Clark*) and *Zea mays* (HiII), respectively. (c) Chiral-HPLC chromatograms showing different epicatechin stereoisomers generated from *in vitro* assays using GmANR1 or ZmANR1 as indicated in (b). SD, standard mixture of (-)-epicatechin and (+)-epicatechin.

Fig. S2

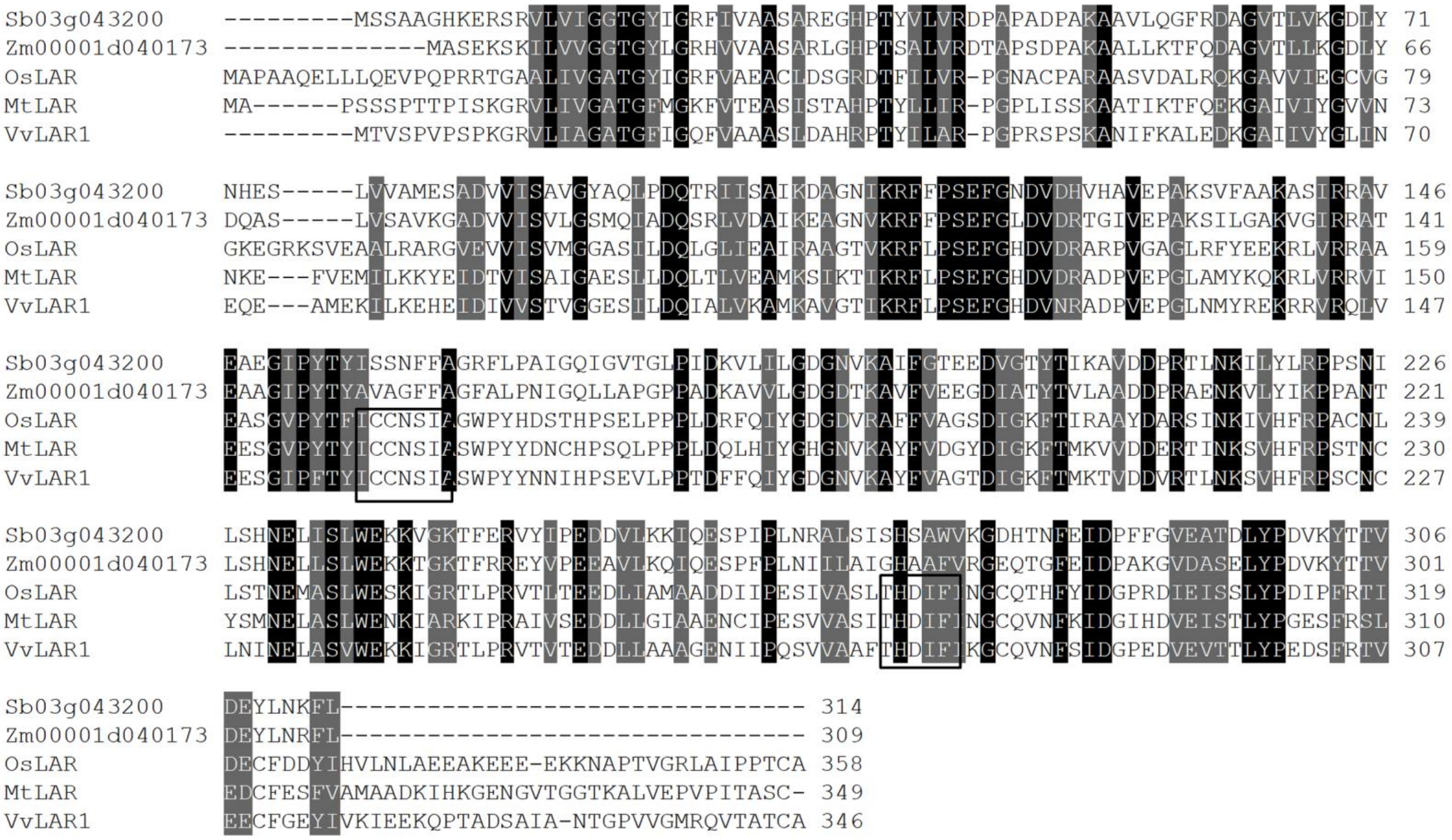

Fig. S2 Amino acid sequence alignment of LAR-like genes from selected monocot and dicot species. GenBank identification numbers for sequences used in the alignment include OsLAR (BN000704.1), MtLAR (XP_003591830.1) and VvLAR1 (NP_001267887.1). The conserved ICCNSIA and THDIFI motifs are highlighted.

Fig. S3

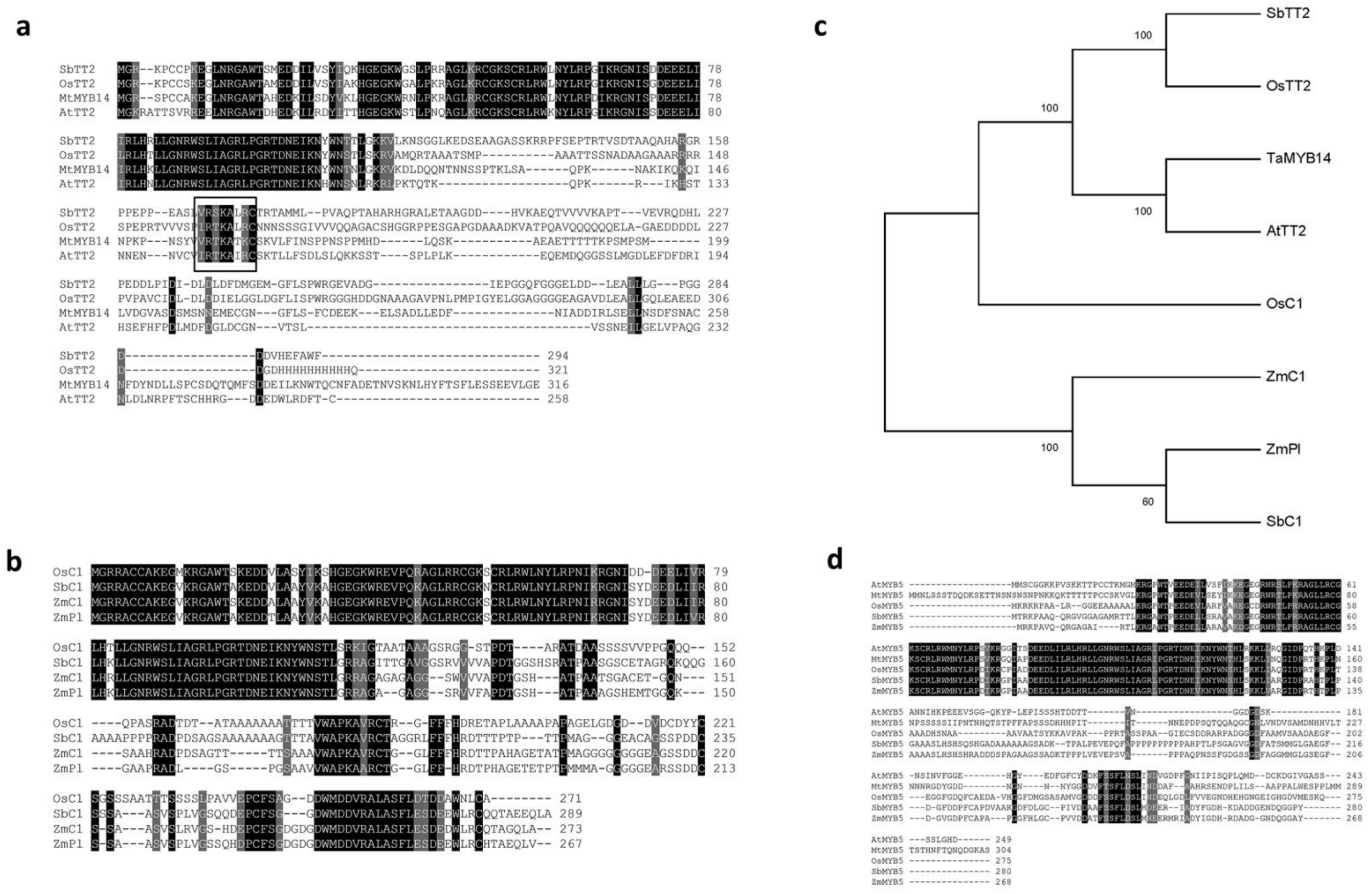

Fig. S3 Identification of TT2-, C1- and MYB5-type MYB transcription factors from monocot and dicot species. (a) Amino acid sequence alignment of TT2-type TFs from sorghum, rice, Medicago and Arabidopsis. The conserved motif for TT2-like TFs is highlighted. (b) Amino acid sequence alignment of ZmC1-type TFs from rice, sorghum and maize. (c) Phylogenetic tree of TT2- and C1-type MYB TFs from sorghum, rice, maize, Arabidopsis and Medicago. (d) Amino acid sequence alignment of SbMYB5, ZmMYB5, AtMYB5, MtMYB5 and OsMYB5. GenBank identification numbers for sequences used to generate the phylogenetic tree include SbTT2 (XP_002465146.1), OsTT2 (BAA23339.1), MtMYB14 (AFJ53057.1), AtTT2 (Q9FJA2.1), OsC1 (XP_015642631.1), SbC1 (XP_002436658.2), ZmC1 (P10290.1), ZmPl (AAB67720.1), AtMYB5 (np187963), MtMYB5 (xp003601609), OsMYB5 (XP_015644524), SbMYB5 (XP_002456198) and ZmMYB5 (XP_008656780).

Fig. S4

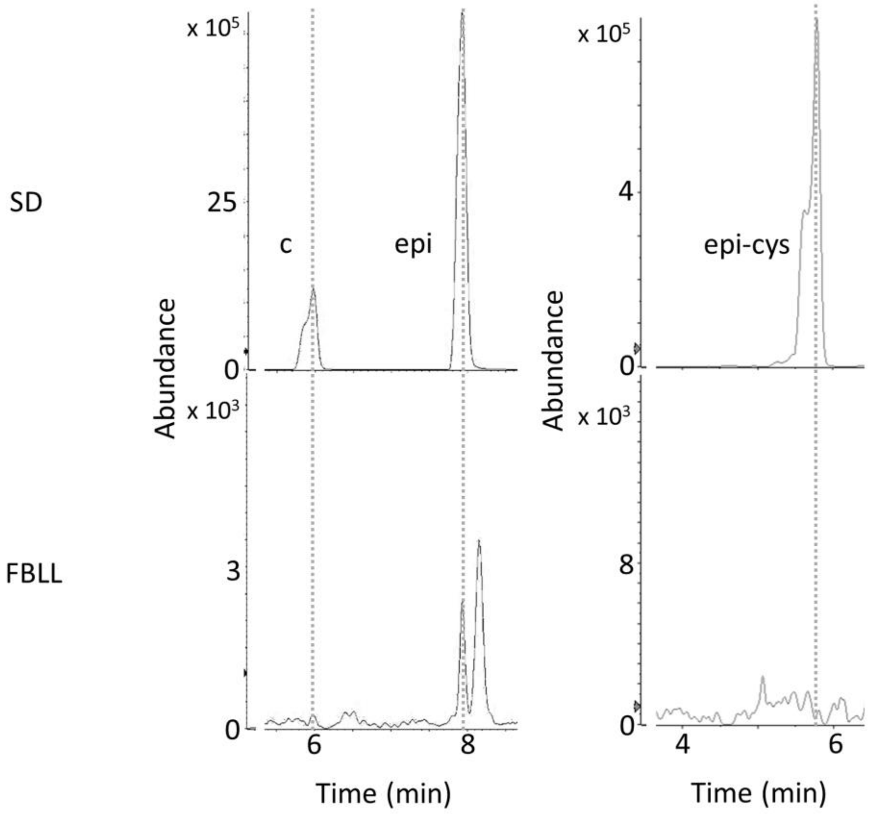

Fig. S4 Analysis of PA monomers in FBLL maize seeds. Selected ion chromatogram of catechin and epicatechin (m/z = 289.0718 ± 10 ppm), as well as 4*β*-(S-cysteinyl)-epicatechin (m/z = 408.0759 ± 10 ppm) in PA extracts from FBLL seeds. SD, chemical standards.

Fig. S5

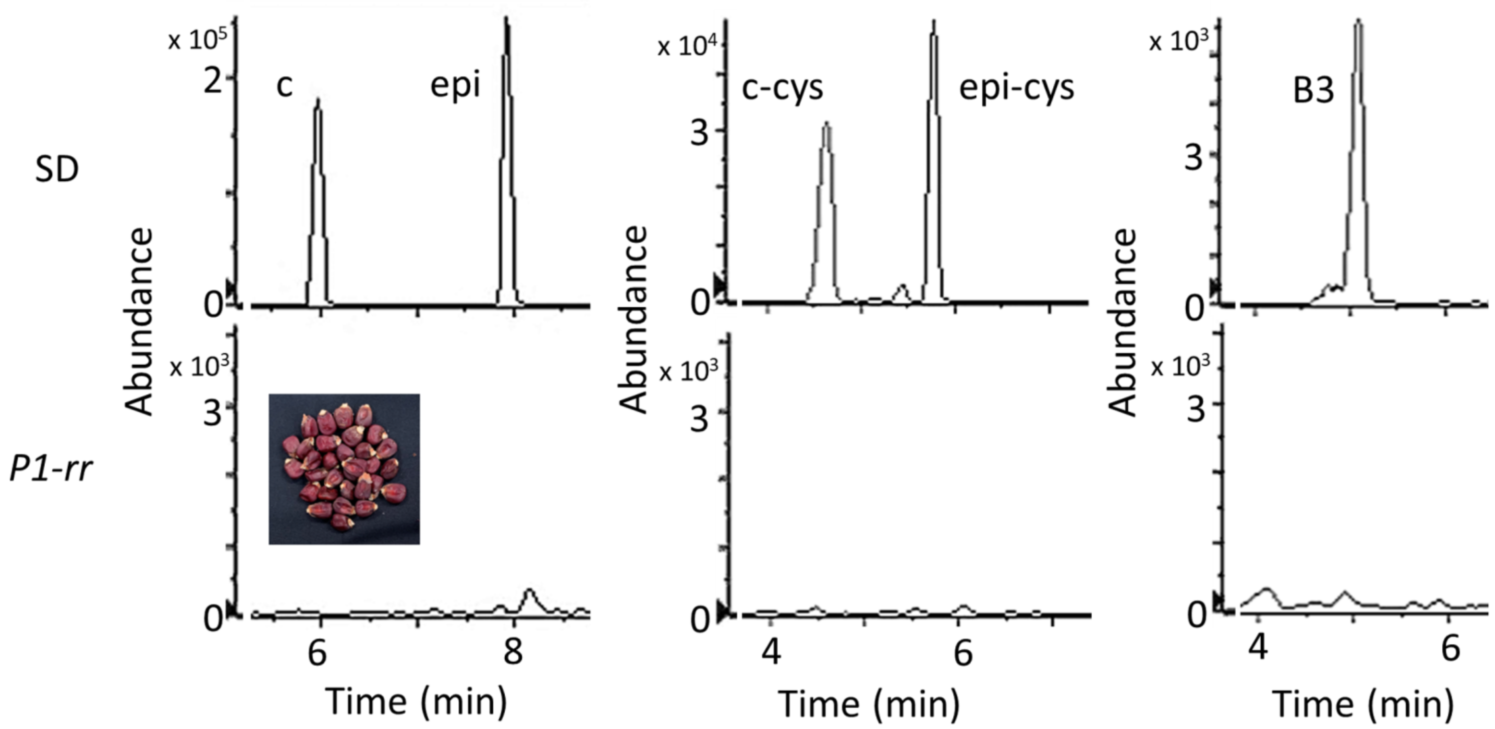

Fig. S5 Examination of the presence of PA precursors in phlobaphene-rich maize seeds. Selected ion chromatograms of catechin and epicatechin (left, m/z = 289.0718 ± 10 ppm), cysteinyl-catechin and cysteinyl-epicatechin (middle, m/z = 408.0759 ± 10 ppm), and procyanidin dimer B3 (right, m/z = 577.1360 ± 10 ppm) in phlobaphene-rich (*P1-rr*) maize seeds. SD, authentic standards (upper panels).

Fig. S6

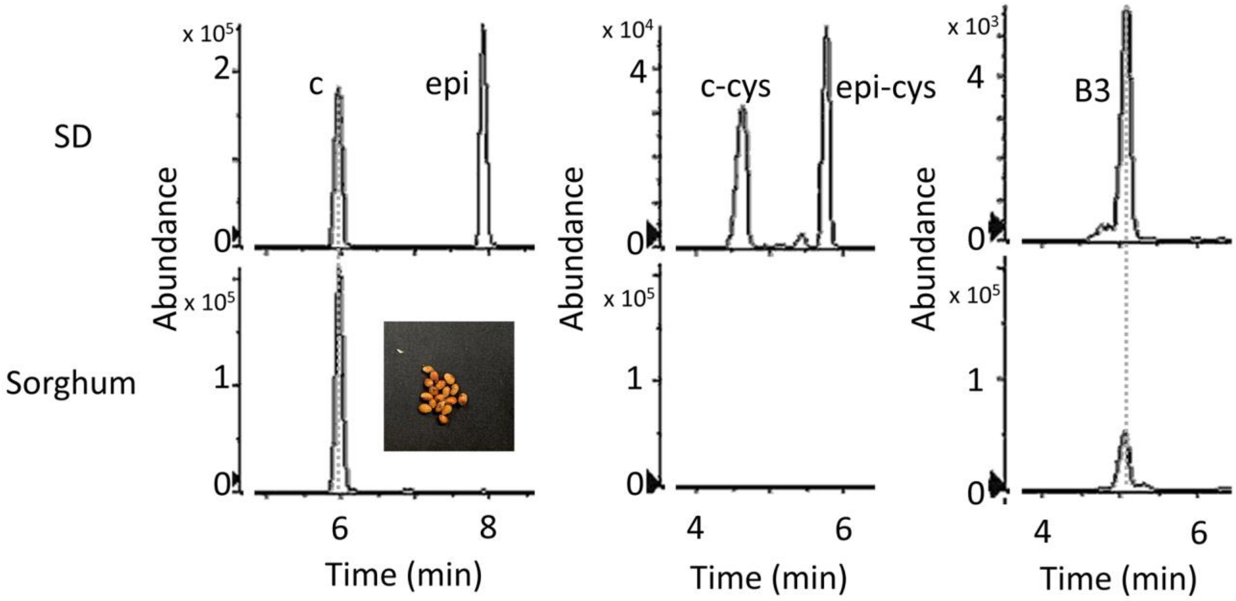

Fig. S6 Examination of the presence of PAs in sorghum. Selected ion chromatograms of catechin and epicatechin (left, m/z = 289.0718 ± 10 ppm), cysteinyl-catechin and cysteinyl-epicatechin (middle, m/z = 408.0759 ± 10 ppm), as well as procyanidin dimer B3 (right, m/z = 577.1360 ± 10 ppm) in sorghum seeds.

Fig. S7

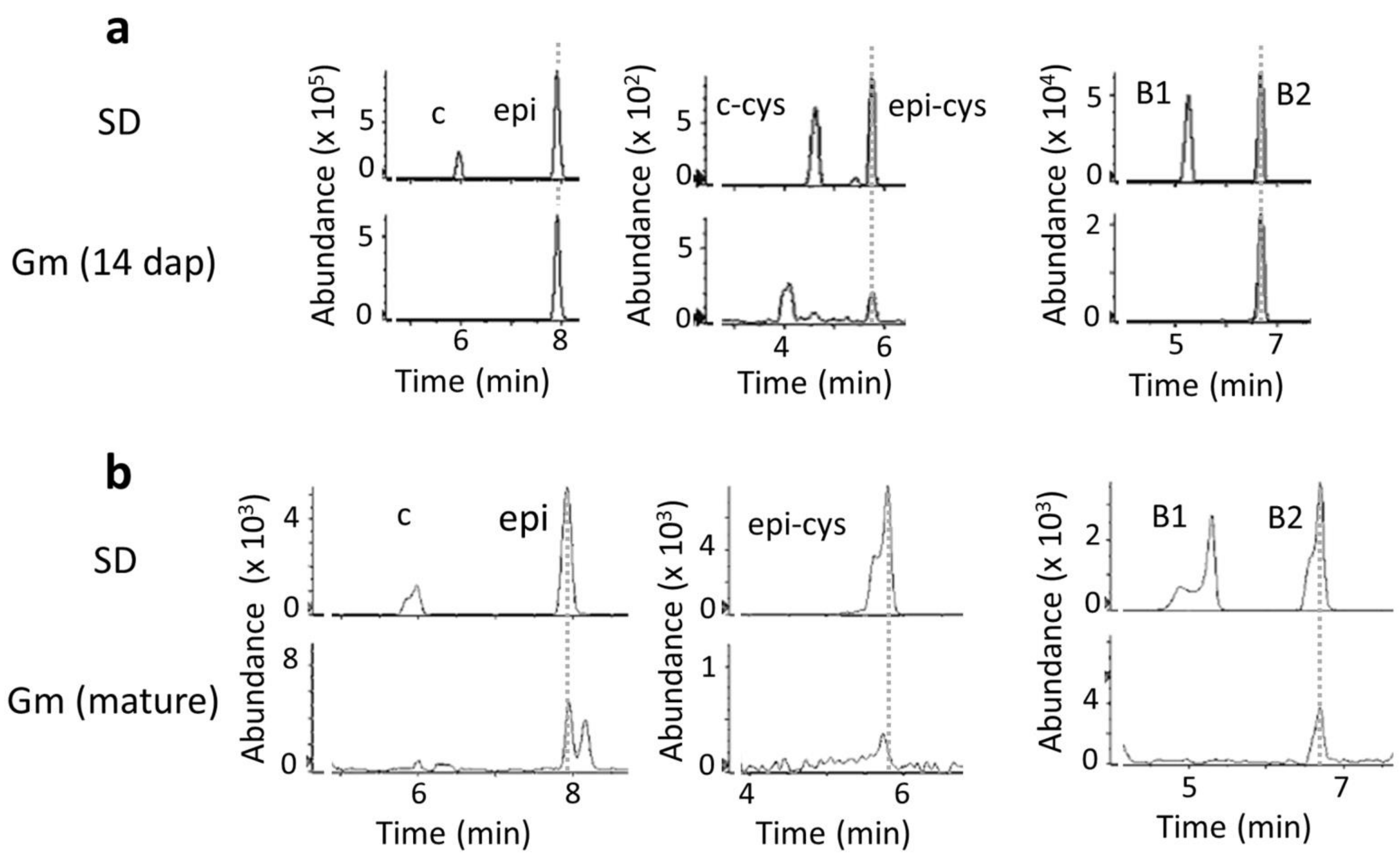

Fig. S7 Analysis of PA monomers and dimers in developing and mature soybean seed coats. Left, selected ion chromatograms of catechin and epicatechin (m/z = 289.0718 ± 10 ppm) in developing (a) and mature (b) soybean seed coats; middle, selected ion chromatograms of 4*β*-(S-cysteinyl)-catechin and/or 4*β*-(S-cysteinyl)-epicatechin (m/z = 408.0759 ± 10 ppm) in developing (a) and mature (b) soybean seed coats; right, selected ion chromatograms of procyanidin dimers B1 and B2 (m/z = 577.1360 ± 10 ppm) in developing (a) and mature (b) soybean seed coats. SD, chemical standards.

Fig. S8

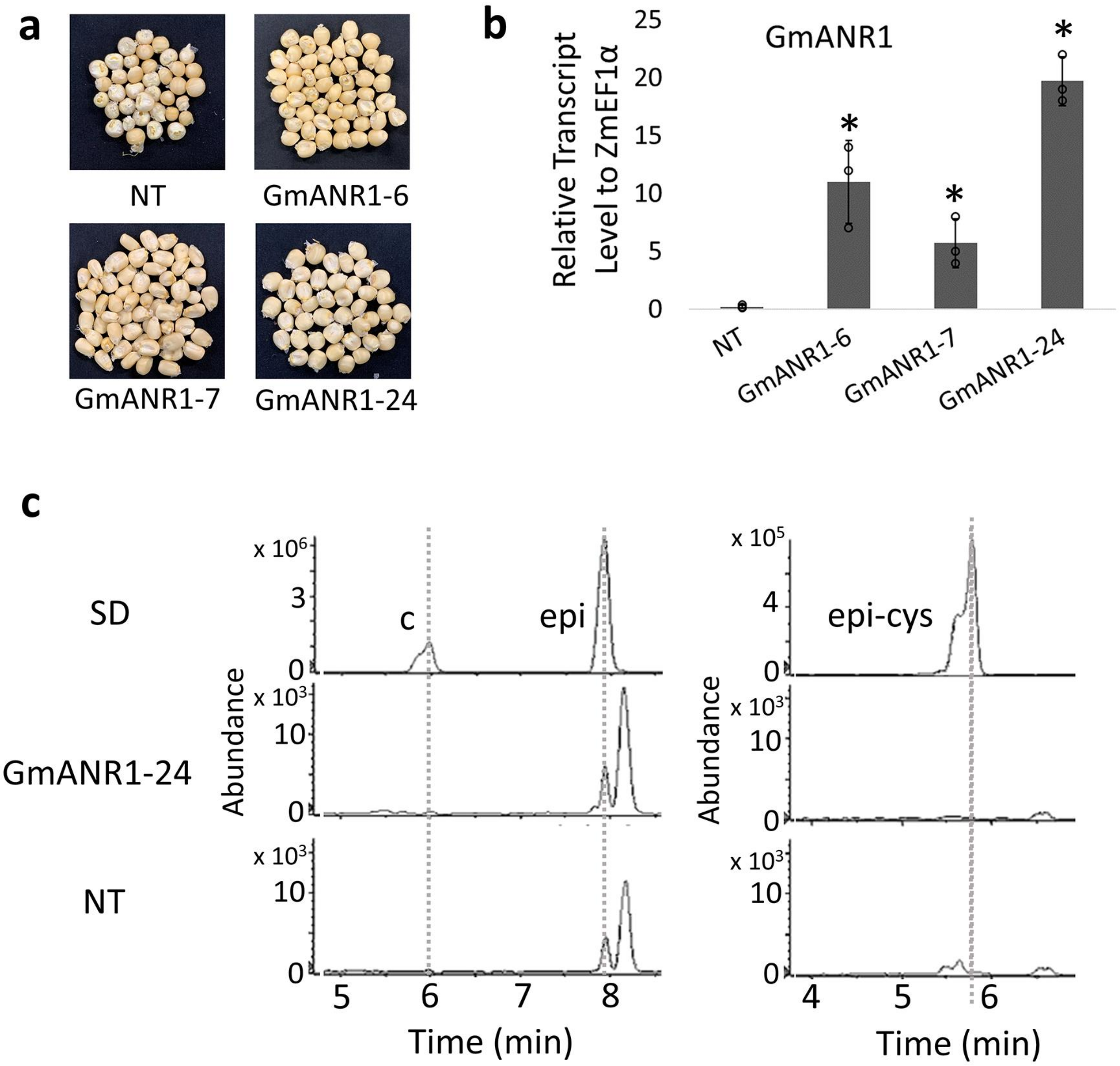

Fig. S8 Expression of GmANR1 does not facilitate PA biosynthesis in HiII maize that does not accumulate anthocyanins. (a) Images of seeds from non-transformed (NT) and three independent transgenic HiII lines expressing *GmANR1* (GmANR1-6, GmANR1-7, GmANR1-24). (b) Transcript levels of *GmANR1* in non-transformed (NT) and transgenic maize analyzed by qRT-PCR. Data are presented as mean ± S.D. (n = 3, independent biological replicates). Asterisks indicate significant difference relative to the non-transformed (NT) control at $P < 0.01$ as determined by two-tailed Student's *t*-test. (c) Selected ion chromatograms of catechin and epicatechin (m/z = 289.0718 ± 10 ppm), as well as 4β-(S-cysteinyl)-catechin and 4β-(S-cysteinyl)-epicatechin (m/z = 408.0759 ± 10 ppm) in non-transformed (NT) and transgenic HiII seeds. SD, chemical standards. Source data for Supplemental Fig. 8b are provided as a Source Data file.

Fig. S9

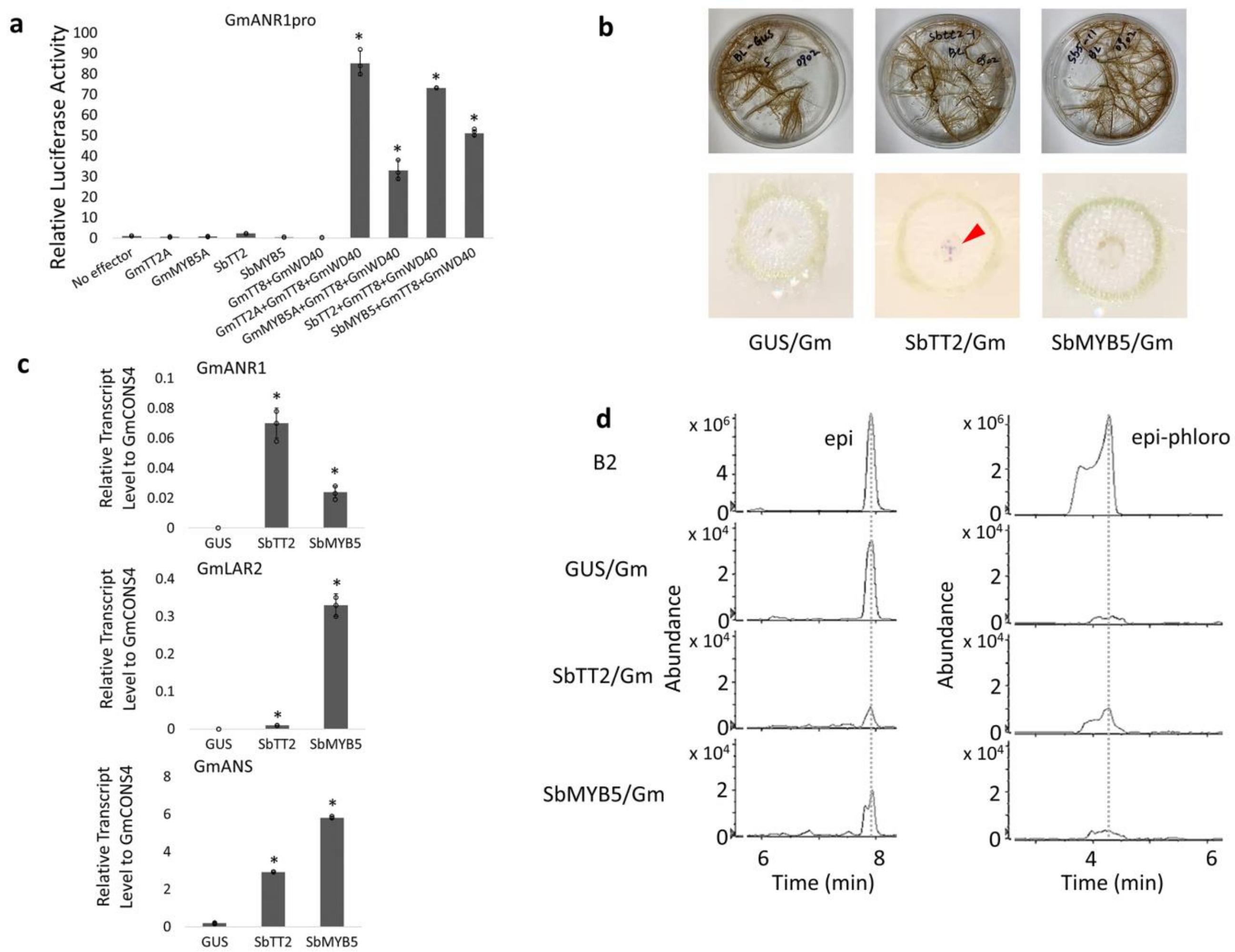

Fig. S9 Characterization of SbTT2 and SbMYB5 using promoter transactivation assays and expression in transgenic soybean hairy roots. (a) Transactivation assays using the GmANR1 promoter in Arabidopsis protoplasts. Effector constructs used for transfection are shown on the X axis. Data are presented as mean ± S.D. (n = 3, independent biological replicates). Asterisks indicate significant difference relative to the negative control at $P < 0.01$ as determined by two-tailed Student's *t*-test. (b) Images of soybean hairy roots expressing *GUS*, *SbTT2* or *SbMYB5* before (top) and after (bottom) DMACA staining. The distribution of PA in soybean hairy root cross sections is shown as purple color after DMACA staining and indicated by the red arrowhead. Images are representative of three independent replicates. (c) Transcript levels of *GmANR1*, *GmLAR2* and *GmANS* in soybean hairy roots expressing *GUS*, *SbTT2* or *SbMYB5*. *GmCONS4* was used as the reference gene. Data are presented as mean ± S.D. (n = 3, independent biological replicates). Asterisks indicate significant difference relative to the GUS control at $P < 0.01$ as determined by two-tailed Student's *t*-test. (d) Phloroglucinolysis of procyanidin B2 standard and PAs extracted from transgenic soybean hairy roots. Selected ion chromatograms of epicatechin (left, m/z = 289.0718 ± 10 ppm) and epicatechin-phloroglucinol (right, m/z = 413.0876 ± 10 ppm) after phloroglucinolysis. Source data for Supplemental Fig. 9a and 9c are provided as a Source Data file.

Fig. S10

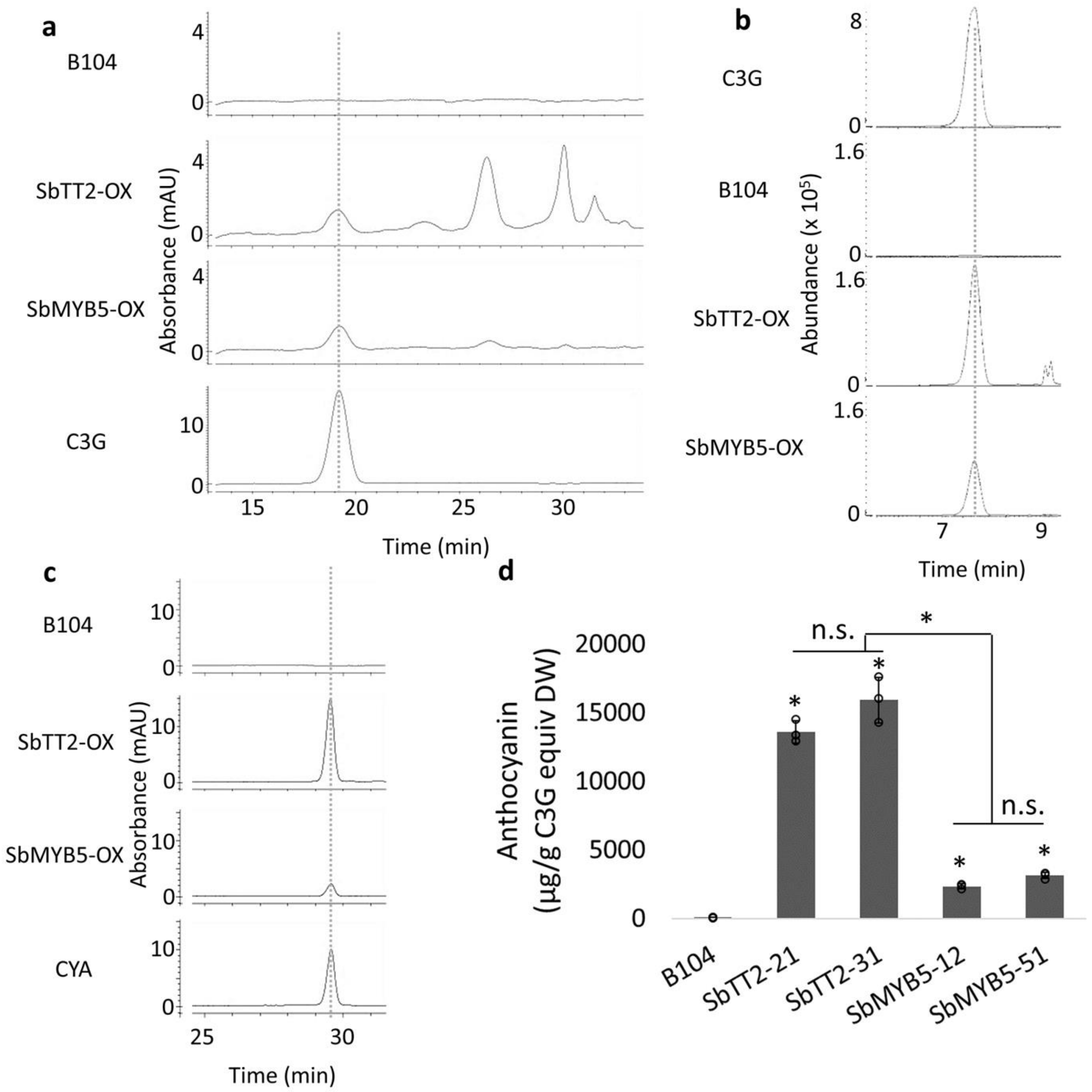

Fig. S10 Analysis of anthocyanins in untransformed and transgenic B104 maize seeds. (a) HPLC chromatograms of anthocyanins extracted from B104, SbTT2-OX and SbMYB5-OX seeds. Cyanidin 3-*O*-glucoside (C3G) was used as the standard. Signals were recorded at 520 nm. (b) Selected ion chromatograms of cyanidin 3-*O*-glucoside (m/z = 447.0940 ± 10 ppm) extracted from SbTT2-OX and SbMYB5-OX seeds. (c) HPLC chromatograms of anthocyanidins released from acid hydrolysis of total anthocyanins extracted from B104, SbTT2-OX and SbMYB5-OX seeds. Cyanidin (CYA) was used as the standard. Signals were recorded at 520 nm. (d) Contents of anthocyanins extracted from B104, SbTT2-21, SbTT2-31, SbMYB5-12 and SbMYB5-51 maize seeds. Cyanidin 3-*O*-glucoside (C3G) was used as the standard. Data are presented as mean ± S.D. (n = 3, independent biological replicates). Asterisks indicate significant difference relative to the untransformed B104 control or between *SbTT2* and *SbMYB5* transgenic lines at $P < 0.01$ as determined by two-tailed Student's *t*-test. Groups with no significant difference were indicated as n.s.. Source data for Supplemental Fig. 10d are provided as a Source Data file.

Fig. S11

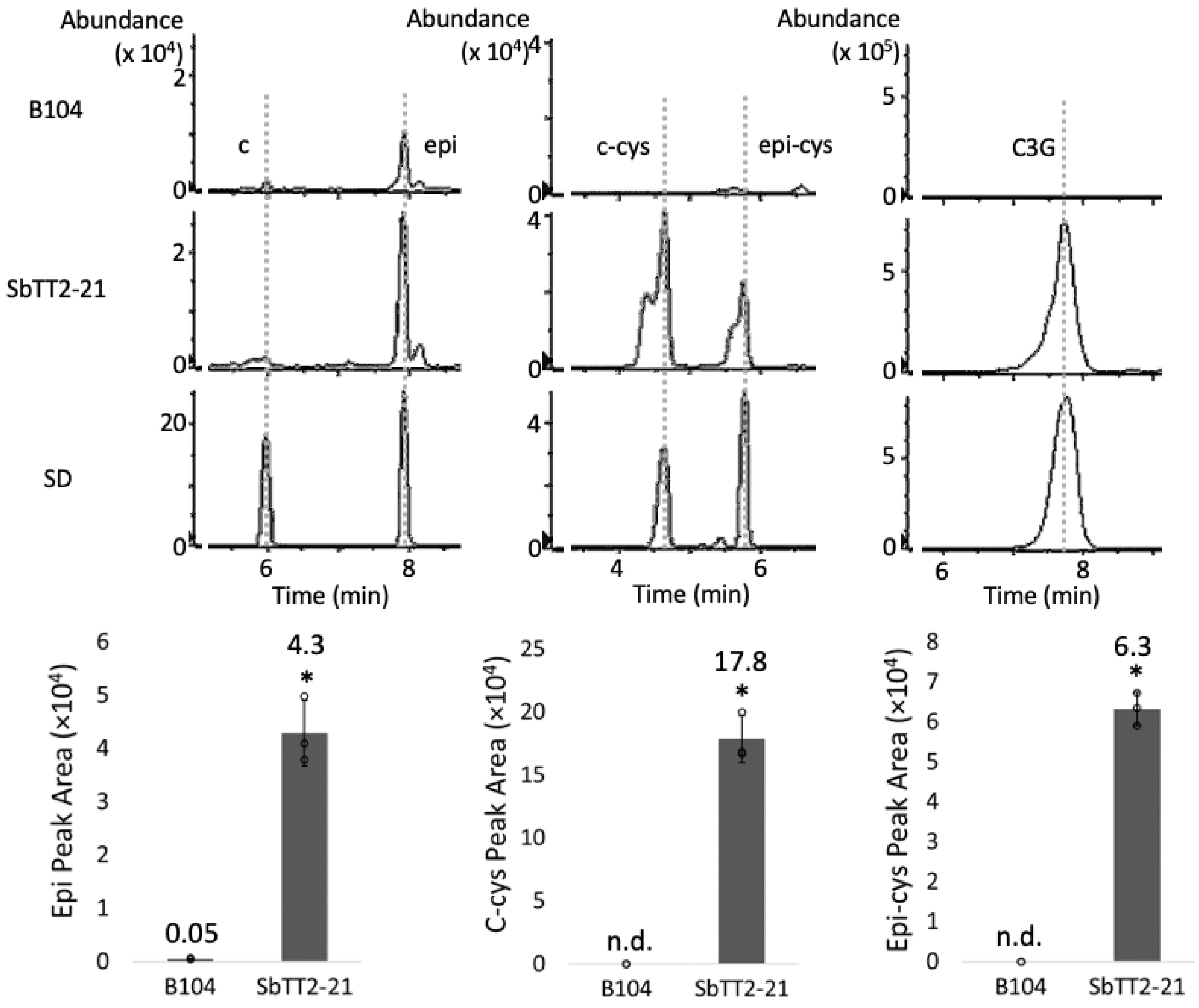

Fig. S11 PA and anthocyanin content in seeds of B104 maize expressing *SbTT2*. Selected ion chromatograms of catechin and epicatechin (left, m/z = 289.0718 ± 10 ppm), 4β-(S-cysteinyl)-catechin and 4β-(S-cysteinyl)-epicatechin (middle, m/z = 408.0759 ± 10 ppm) and cyanidin 3-*O*-glucoside (right, m/z = 447.0940 ± 10 ppm) in seeds of B104 and SbTT2-OX (line SbTT2-21). Peak areas of epicatechin (epi), 4β-(S-cysteinyl)-catechin (c-cys), and 4β-(S-cysteinyl)-epicatechin (epi-cys) in B104 and SbTT2-OX were shown in histograms. Data are presented as mean ± S.D. (n = 3, independent biological replicates). The numbers on top of bars represent average peak area value. Asterisks denote significant difference relative to untransformed B104 at $P < 0.01$ determined by two-tailed Student's *t*-test. Compounds not detected are labeled as n.d.. Source data for Supplemental Fig. 11 are provided as a Source Data file.

Fig. S12

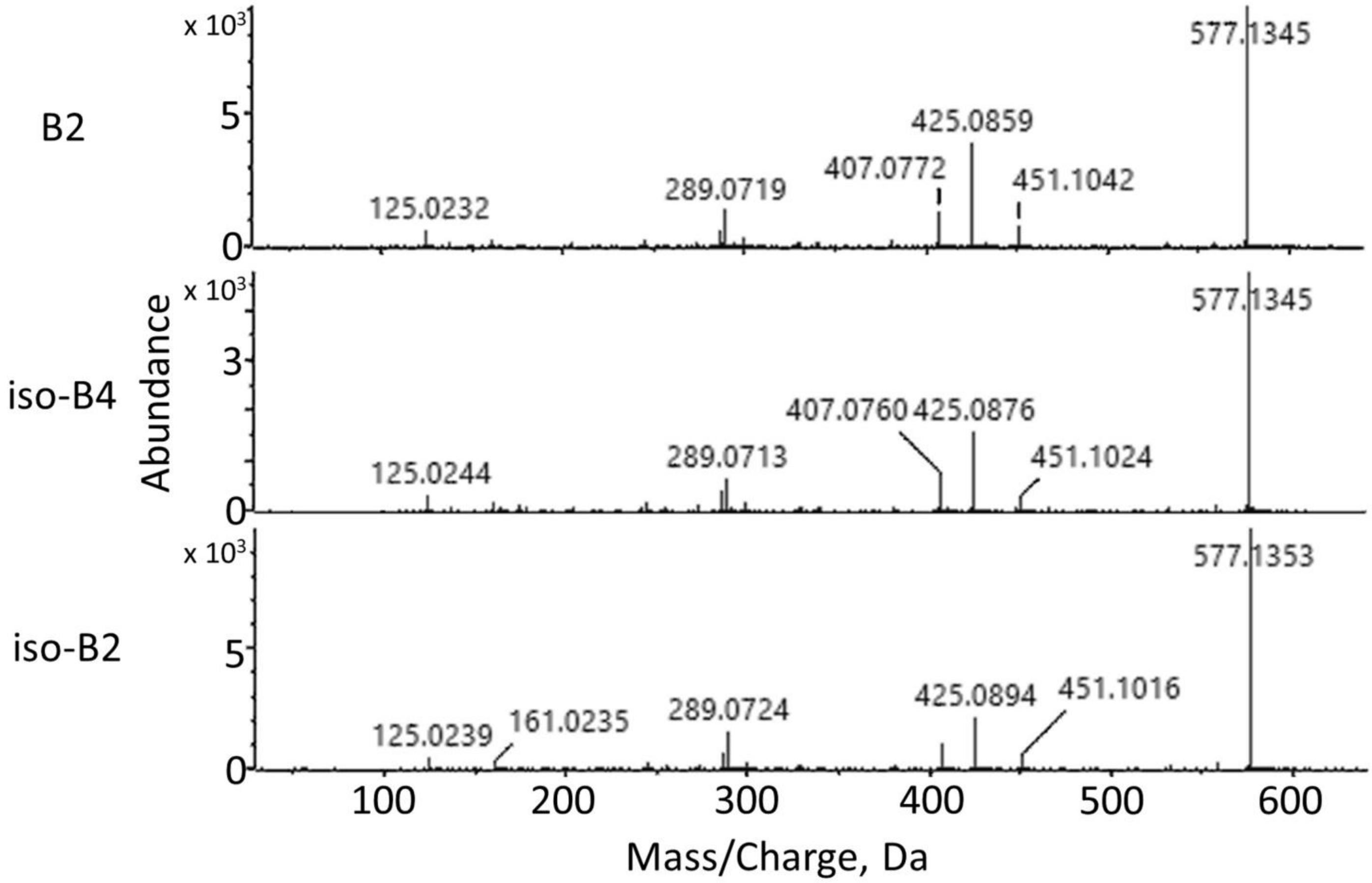

Fig. S12 Mass spectra of procyanidin dimers analyzed by LC-MS in this study. MS/MS spectra of procyanidin dimers B2, iso-B4 and iso-B2.

Fig. S13

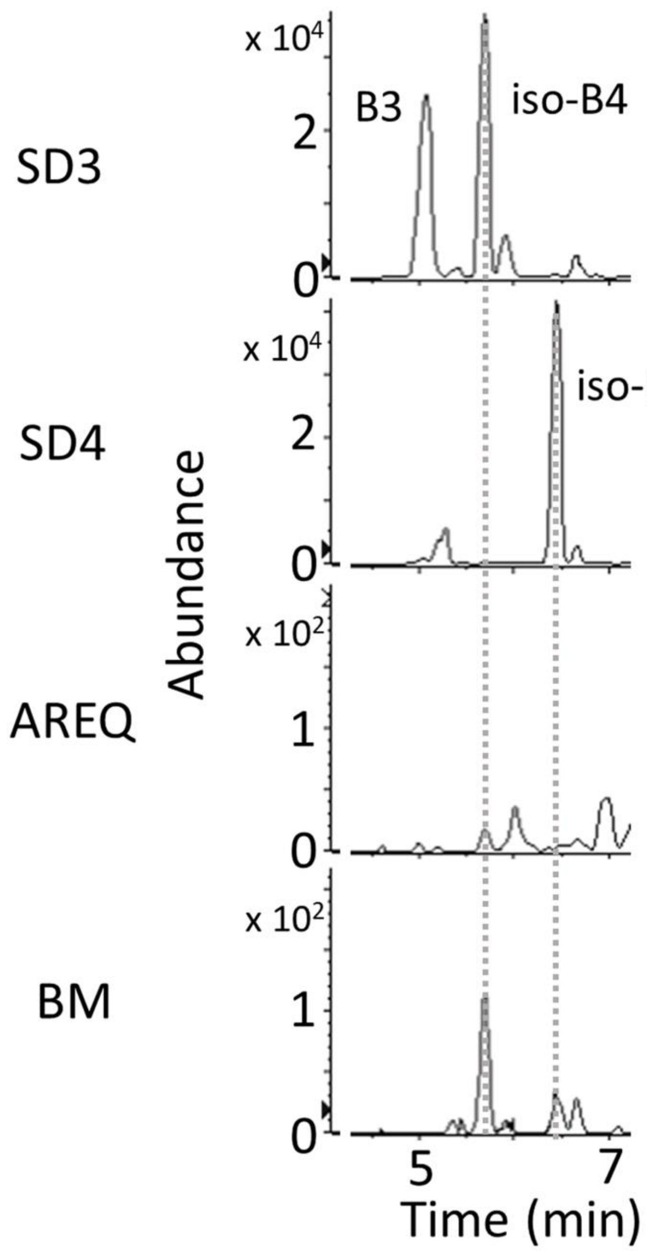

Fig. S13 Analysis of procyanidin dimers in AREQ and BM maize seeds. Selected ion chromatograms of procyanidin dimers (m/z = 577.1360 ± 10 ppm) in AREQ and BM maize seeds. SD, chemical standards.

Fig. S14

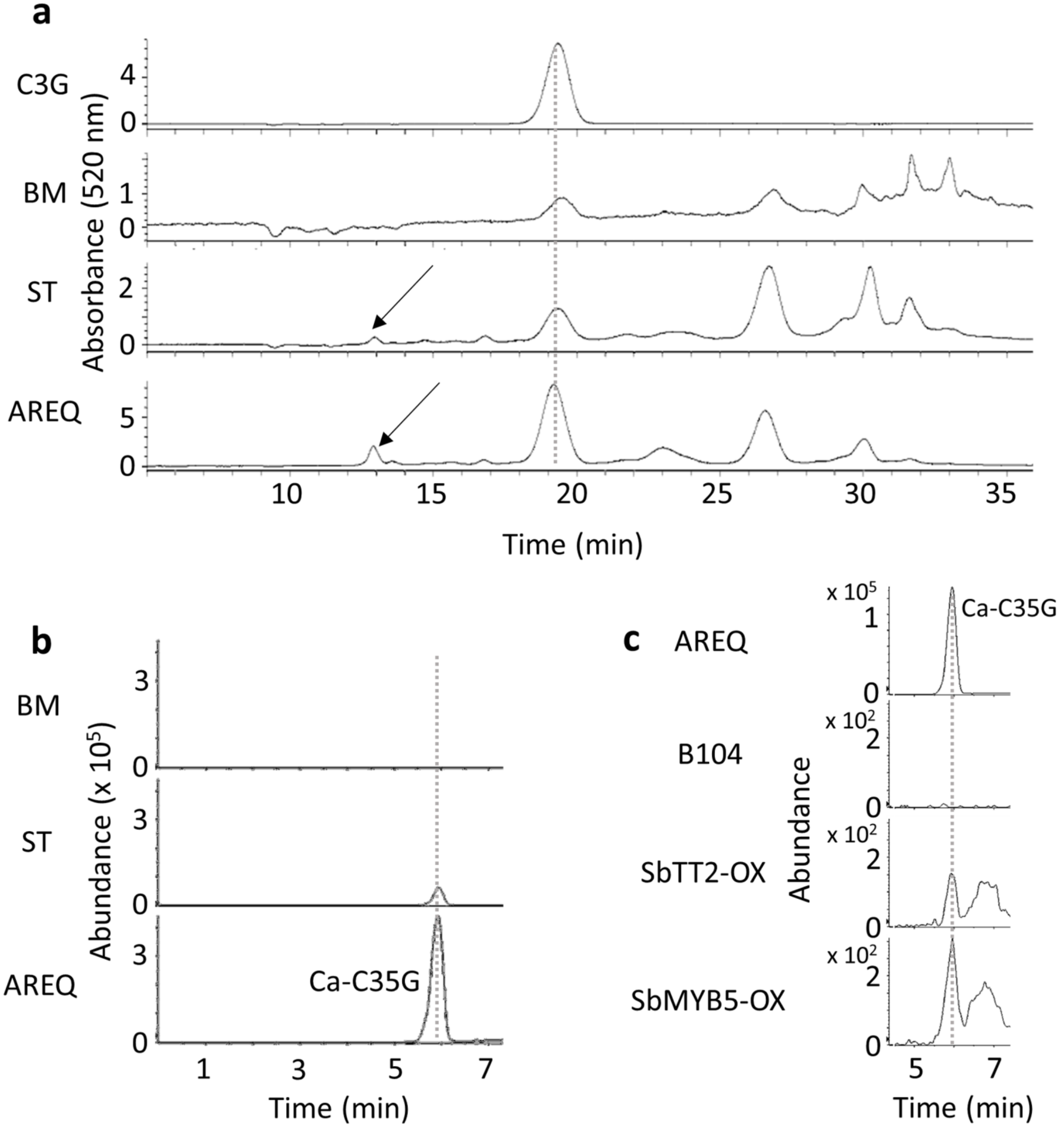

Fig. S14 Analysis of flavanol-anthocyanin conjugates in maize seeds. (a) HPLC chromatograms of total anthocyanins extracted from Black Mexican (BM), ST (Suntava) and Arequipa (AREQ) maize seeds. C3G, cyanidin 3-*O*-glucoside. Signals were recorded at 520 nm. Arrows indicate flavanol-anthocyanin conjugates. (b) Selected ion chromatograms of catechin-cyanidin-3,5-diglucoside (Ca-C35G, m/z = 897.2127 ± 10 ppm) in ST and AREQ seeds generated by LC-MS. (c) Selected ion chromatograms of Ca-C35G (m/z = 897.2127 ± 10 ppm) in transgenic seeds expressing *SbTT2* or *SbMYB5* generated by LC-MS.

Fig. S15

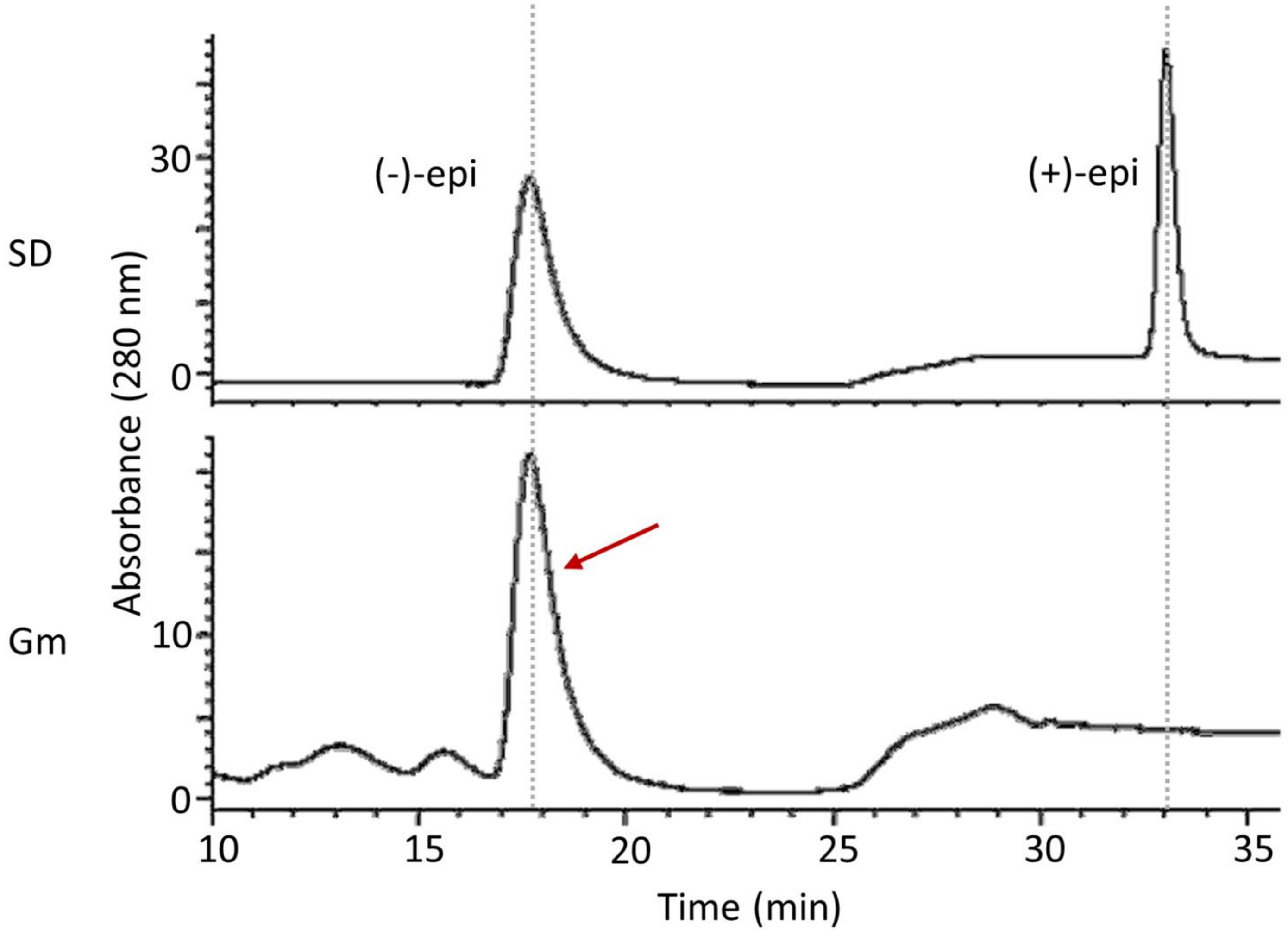

Fig. S15 Chiral HPLC analysis of stereochemistry of epicatechin extracted from soybean seed coats. Chiral HPLC chromatograms of PAs extracted from soybean seed coats. The (-)-epicatechin in PAs extracted from soybean (Gm) seed coat is indicated by the red arrow. SD, chemical standard; (-)-epi and (+)-epi, (-)-epicatechin and (+)-epicatechin standards. Signals were recorded at 280 nm.

Fig. S16

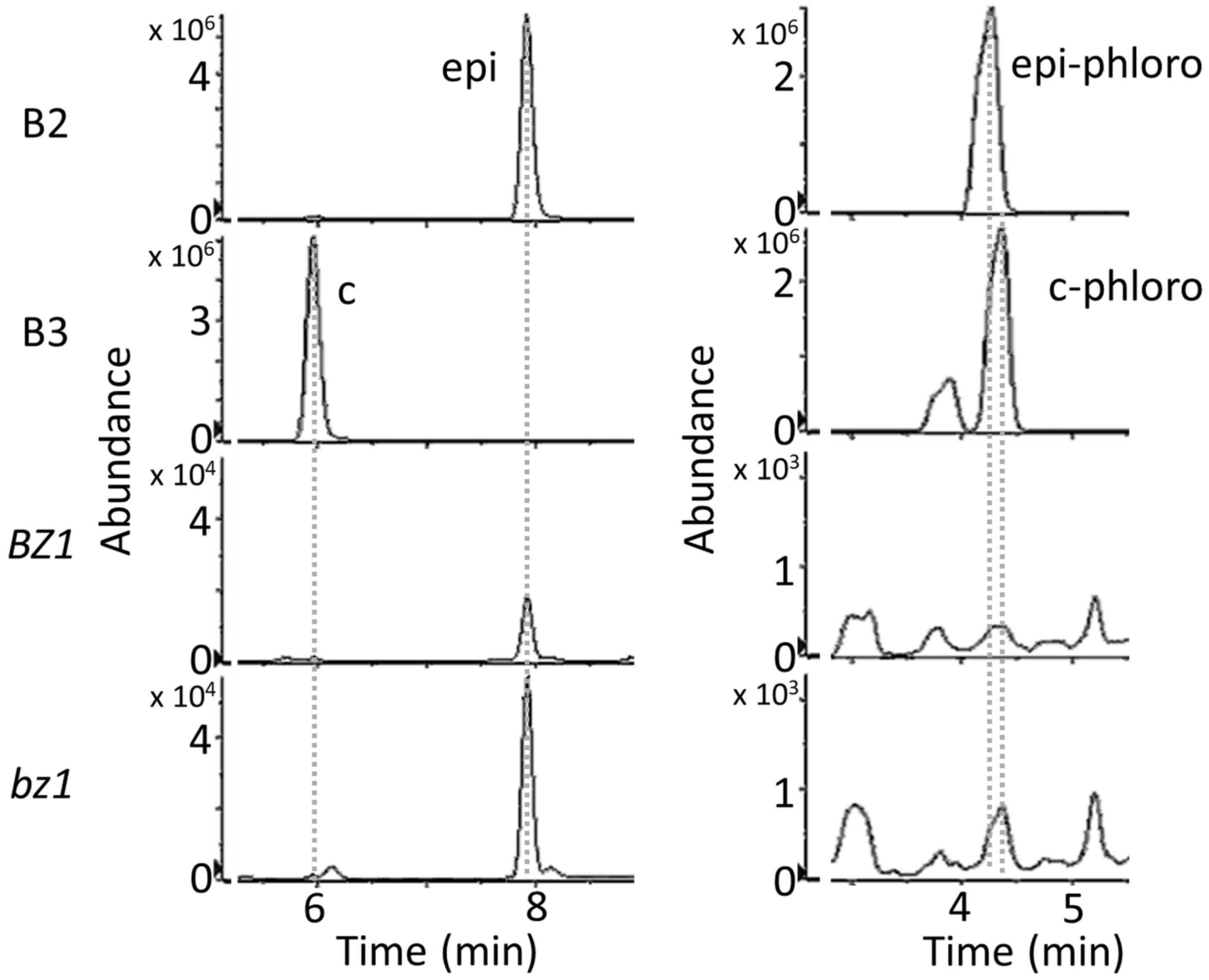

Fig. S16 Phloroglucinolysis of PAs extracted from *BZ1* and *bz1* maize seeds. Selected ion chromatograms of (epi)catechin (left, m/z = 289.0718 ± 10 ppm) and (epi)catechin-phloroglucinol (right, m/z = 413.0876 ± 10 ppm) after phloroglucinolysis. Procyanidin B2 and B3 were used as standards.

Primer ID	Primer sequence (5'-3')
ZmEF1aF	TGGGCCTACTGGTCTTACTACTGA
ZmEF1aR	ACATACCCACGCTTCAGATCCT
ZmANR1qRTF	CATCTGCTGCGGCCTCAACAC
ZmANR1qRTR	CACCCTCACCCTCGGCTTCT
ZmANR2qRTF	CGAACCTGCTCTCCGGCGAG
ZmANR2qRTR	TGTCATCCAGCGTCCTGTACCTG
SbTT2qRTF	TTGGAGACAGCTGCGGGCGA
SbTT2qRTR	TCAGGAAACCCATCTCACCCATG
SbMYB5qRTF	GGTTCTGCCGACAAGACGCC
SbMYB5qRTR	AATCCATCGAAGCCCTCGGCG
GmCONS4F	GATCAGCAATTATGCACAACG
GmCONS4R	CCGCCACCATTCAGATTATGT
GmANR1qRTF	CCCTCAATATAAAATTCCAACTGAA
GmANR1qRTR	CAGAGCCCCTTTGCTCTTTA
GmLAR2qRTF	GGTGTGATAAATAACAAGGACTTTGTGG
GmLAR2qRTR	CTCTGAAGGCAAAAACCTCTTAATAGTC
GmANSqRTF	CTGAGCAACGGCAAGTACAA
GmANSqRTR	CAATTTGGGAGACCTTCCTG
AtANRqRTF	ACCCAATCTCGAAGGTGTTAG
AtANRqRTR	GGAGAGAGTTTCCGGCTATAAG
NtEF1aF	TGAGATGCACCACGAAGCTC
NtEF1aR	CCAACATTGTCACCAGGAAGTG

Table S1. Primers used in the present work.
